# Haemophilia - diagnosis and management challenges

**DOI:** 10.1186/1755-8166-7-S1-I44

**Published:** 2014-01-21

**Authors:** Shrimati Shetty

**Affiliations:** 1 ICMR-National Institute of Immunohaematology Parel, Mumbai, India

## 

Haemophilia care in India is slowly progressing but the diagnostic and management challenges continue until optimum care for haemophilia patients become a reality in this country. Against an anticipated severe haemophilia population of more than 0.1 million, only 16000 haemophilia patients are registered in the 74 Haemophilia Chapters across the country. As per the recent WFH Survey, 43% of the World haemophilia population live in India, Bangladesh, Indonesia and China, out of which only 12% is diagnosed in these countries. Additionally these countries represent only 2% of the Worlds total factor usage. The per capita use of FVIII in India has been shown to be 0.0075 (1IU per capita is 20000 IU FVIII per haemophilia patient) which is approximately equal to mean consumption of 1654 IUs per PWH against 112508 IUs per PWH in United States. Haemophilia in India is thus a grossly under diagnosed disorder, mainly due to lack of awareness and diagnostic facilities in this country.

Except a few Corporate Hospitals and a few Medical Colleges in India majority of the 630 District hospitals and Medical Colleges do not have even screening coagulation facilities. Haemophilia Federation (India) has taken wide initiatives in providing diagnostic facilities to the PWH in this country, through various measures such as supporting the cost of diagnosis of PWH, networking private laboratory facilities, setting up coagulation laboratories in close proximity to Haemophilia Chapters, involving faculty members of Medical Colleges in the activities of Haemophilia Chapters and so on. Indian Council of Medical Research has also initiated through its Translation Research Programme several workshops in the Laboratory diagnosis of bleeding disorders at Mumbai and several places in East and North Eastern parts of India. As quality control is an important exercise in a coagulation laboratory, many of the laboratories in India fail to establish factor and inhibitor screening assays in their respective laboratories.

The prevalence of inhibitors in India is estimated to be 8.2–13%. Post operative inhibitors are another important problem which our PWH is facing in India. About 30% of PWH undergoing surgeries for various indications develop inhibitors postoperatively. Diagnosing inhibitors, demands specialized laboratories and expertise. The management of PWH and inhibitors comprises several approaches involving prompt treatment of bleeding episodes, managing its complications, preventing bleeds, and conserving and restoring joint function. The ultimate goal of treating PWH and inhibitors is to permanently eradicate inhibitors by immune tolerance therapy (ITT), or by use of alternative products like rFVIIa or FEIBA. However, because of prohibitive cost and logistics constraints many of our PWH are not able to utilize these products. Availability of free factors in some states of our country is a refreshing and welcome change for PWH. Significant progress has been made in awakening and alerting the State Governments for providing free factors to all our PWH. WFH has contributed to haemophilia care in India through a series of international twinning programme by initially twinning upcoming chapters in India or institutions of repute in India with some of the best centres in Europe and USA leading to the development of expertise in the area of genetic diagnosis and physical therapy.

Despite this, there are several newer challenges that the hemophilia community will face in the coming years. Normalization of patients' lives as a result of improved treatment has led to new problem areas. Malignancies specifically hepatocellular carcinoma (HCC) in HCV infected patients and non Hodgkin’s lymphoma in HIV infected patients are on the rise. About 25% of our patients is HCV infected. A less common albeit potentially severely morbid event is ICH. Osteoporosis in general has been considered to be an important cause of morbidity in patients with haemophilia and other bleeding disorders.

